# Generalized Lymphadenopathy as the First Presentation of Granulocytic Sarcoma: A Diagnostic Challenge

**DOI:** 10.1155/2013/483291

**Published:** 2013-10-27

**Authors:** Ghaleb Elyamany, Mohammed Khan, Imad El Hag, Maha El-Zimaity, Mohamed Albalawi, Abdulaziz AL Abdulaaly

**Affiliations:** ^1^Department of Hematology and Blood Bank, Theodor Bilharz Research Institute, Egypt; ^2^Hematopathology, Prince Sultan Military Medical City, P.O. Box 7897, Riyadh 11159, Saudi Arabia; ^3^Department of Central Military Laboratory, Histopathology Division, Prince Sultan Military Medical City, Saudi Arabia; ^4^Department of Adult Clinical Hematology and Stem cell Therapy, Prince Sultan Military Medical City, Saudi Arabia

## Abstract

*Introduction*. Granulocytic sarcoma (GS), also known as chloroma or extramedullary myeloblastoma, is a solid tumor composed of primitive precursors of the granulocytic series that include myeloblasts, promyelocytes, and myelocytes. Granulocytic sarcoma is a rare tumor that may develop during acute myeloid leukemia (AML) but less frequently may precede its presentation. Although generalized lymph node enlargement is a presentation for malignant lymphoma, it can also rarely be the early presenting sign of GS. *Methods*. We present a case of GS mimicking lymphoma in a 45-year-old male. The patient presented with bilateral neck masses and had widespread, prominent lymphadenopathy secondary to AML as the first presenting manifestation of GS for the last 4 months with concurrent marrow AML. *Result*. A clinical diagnosis of lymphoma was suspected; fine needle aspiration cytology findings were also suggestive of lymphoma. However, peripheral blood and bone marrow examination reported as acute myeloid leukemia with monocytic differentiation and histopathology of excised lymph node confirmed it to be a GS not lymphoma. *Conclusion*. GS is often misdiagnosed as malignant lymphoma because of cytomorphologic and histologic similarities of the blasts to large cell lymphoma. A careful search for immature myeloid is a useful clue to the diagnosis accompanied with appropriate immunophenotyping.

## 1. Introduction

Granulocytic sarcoma (GS) or myeloid sarcoma is a unique rare entity. In early reports, GS was known as chloroma, because of its rich myeloperoxidase content that appeared green. GS is a solid tumor composed of immature cells of the granulocyte series [1, 2]. Most GS present with multiple masses involving any part of the body [3]. These tumors may develop during or as a presenting sign of myelogenous leukemia but may precede acute myelogenous leukemia (AML) by months or years or represent the initial manifestation of relapse in a previously treated AML in remission [4, 5].

GS may herald leukemic transformation in myelodysplastic disorders or myeloproliferative neoplasms, including chronic myeloid leukemia, polycythemia rubra vera, myelofibrosis, and chronic eosinophilic leukemia [6, 7]. The incidence of myeloid sarcoma is 2.5 to 9.1% of the patients with AML and it is five times less frequent in patients with chronic myeloid leukemia. There is predilection for males with male and female ratio of 1.2 : 1 [8]. Skin, lymph node, gastrointestinal tract, brain, bone, soft tissues, and testis are more frequently affected.

The major differential diagnosis is with malignant lymphoma, and distinction of granulocytic sarcoma from lymphoblastic lymphoma, Burkitt lymphoma, diffuse large B-cell lymphoma, small round cell tumor in children, and blastic plasmacytoid dendritic cell neoplasm needs proper immunohistochemical studies [5].

 Although generalized lymph node enlargement is a presentation for malignant lymphoma, it can also rarely be the first presenting sign of GS. The present study describes a case of GS with generalized lymphadenopathy as an early manifestation of acute AML.

## 2. Case Report

This is a 45-year old gentleman who presented with swelling of both sides of his neck for 4 months, followed by swellings in his both armpits. This was associated with on and off fever and sweating. He sought medical advice and received several courses of antibiotics with no response. On examination, the patient had stable vital signs with temperature of 37°C and BP 150/70 mmHg. There was generalized bilateral lymphadenopathy involving preauricular, occipital, axillary, and inguinal lymph nodes. The lymph nodes were not painful or tender with variable sizes reaching up to 8 × 5 × 4 cm. Patient also had mild splenomegaly. Initial workup showed a WBC of 3.3 × 10^9^/L with monocytosis 1.5%, hemoglobin 9.1 gm/dl, platelets 158 × 10^9^/L, LDH 684 U/L, and CRP 186 mg/L with normal liver and renal functions. Initial viral screen was negative for EBV, HIV, HBV, and HCV.

 Computed tomography (CT) scan showed bilaterally enlarged cervical, axillary, hilar, and intraparotid lymph nodes with bulky palatine tonsils and mediastinal lymph nodes. In addition, multiple enlarged lymph nodes were seen at the base of the neck on both sides. There were large lymph nodes in aortopulmonary window, intra-abdominally (para-aortic, retrocaval, portahepatis, mesentery, and along the common iliacs and external iliacs) and within the inguinal region. The largest lymph node seen within the right axilla measured 8 × 5 cm. Most of these lymph nodes showed homogenous moderate density with mild homogenous enhancement. A few nodes in axillae and inguinal regions showed homogenous low-density appearance. The spleen measured 14 cm in length, but no focal lesions were identified. No hepatomegaly was appreciated and no focal lesions were seen. The patient had fluctuating body temperature, the highest reaching 38°C. He was started empirically on Augmentin and Tazocin. After the diagnosis was established as GS, the patient went to another hospital to start chemotherapy.

## 3. Bone Marrow Examination and Flow Cytometry Analysis 

**Figure 1:**
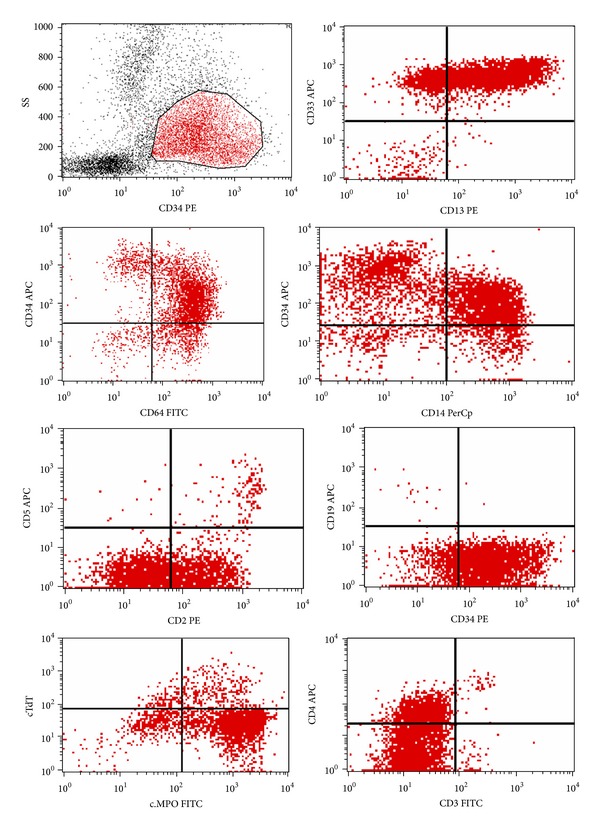
Flow cytometry analysis on PB samples using side scatter (SS) and CD34 positive cells. The blasts showed positivity to CD34, CD33, CD13, CD14, CD64, MPO, CD2, and CD4 and negativity to CD19, CD3, CD5, and TdT. MPO: myeloperoxidase. TdT: terminal deoxynucleotidyl transferase.

The peripheral blood smears revealed circulating blast cells exhibiting features of myeloblasts, monoblasts, promonocytes, and increased numbers of mature monocytes. Flow cytometry (FCM) performed on the peripheral blood (PB) sample revealed a picture of acute myeloid leukemia with a monocytic component (FAB: M4-M5) with aberrant expression of CD2 and CD56 (Figure 1). 

Bone marrow (BM) examination and FCM on BM sample revealed that around 80% of BM cells are blast cells. The blast cells are positive for myeloid markers (CD13, CD33, MPO, and HLA-DR), monocytic markers (CD14, CD64, and CD11c), and immature precursor markers (CD34 and CD117). They were also positive for CD56 and CD2. B-cell and other T-cell markers are negative. The final diagnosis was reported as AML with monocytic differentiation with aberrant expression of CD2 and CD56. 

## 4. Fine Needle Aspirate Cytology

Fine needle aspirate of cervical lymph node showed numerous atypical large lymphoid cells and scattered tingible body macrophages in the background; findings were suggestive of lymphoma. 

## 5. Histological and Immunohistochemical Findings 

**Figure 2:**
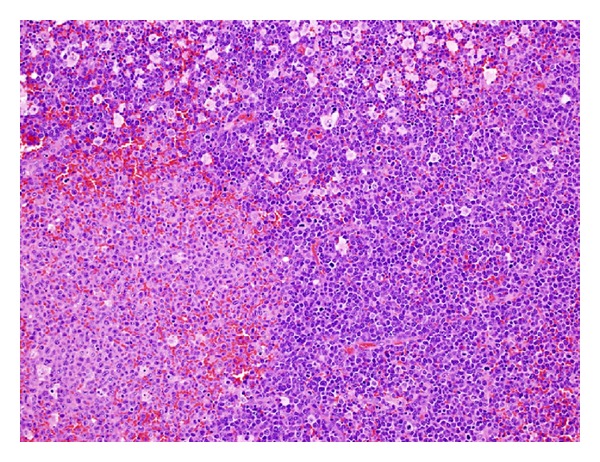
A low power view, displaying a characteristic tumor growth pattern, with pale areas surrounded by darkly staining areas, the latter imparting a starry sky appearance.

**Figure 3:**
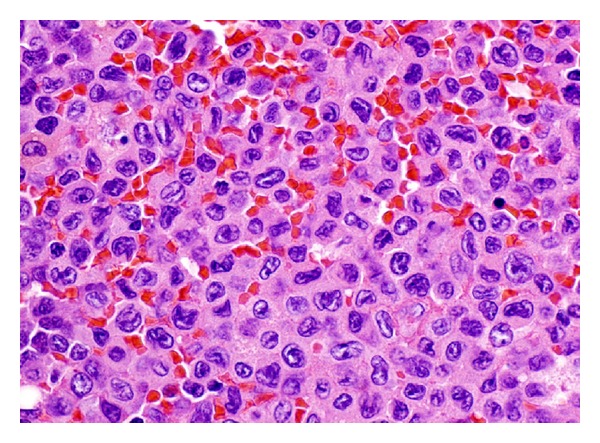
A high power view of the pale areas in Figure 1 showing tumor cells with folded, cleaved, and kidney-bean shaped nuclei.

**Figure 4:**
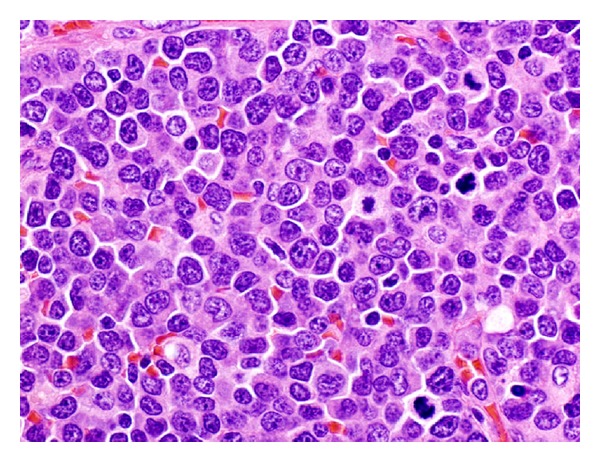
A high power view of darkly staining cellular areas in Figure 1, exhibiting a polymorphous infiltrate, containing cells with large atypical nuclei, with abnormal mitotic figures and scattered apoptotic bodies.

Histological examination of the left axillary lymph node excision biopsy revealed complete effacement of the normal lymph nodal architecture by a diffuse polymorphic abnormal cells infiltrate comprising atypical medium to large sized mononuclear cells with frequent mitoses, many apoptotic cells, and histiocytes, imparting a starry sky pattern in the background (Figure 2). There are distinct pale staining areas composed of abnormal kidney-bean shaped cells with folded or cleaved nuclei (Figure 3). There are adjacent darkly staining areas with atypical large cells displaying a wide spectrum of cellular atypia and pleomorphism. The residual small lymphoid follicles are also present (Figure 4).

Most of the atypical cells show a myeloid origin and show positive immunostaining for CD33, CD34, and CD117 and focal immunostaining for MPO, CD123, CD163, and CD68 but negative for CD14. There is a separate population of atypical cells showing a more primitive T-cell immunophenotype characterized by positive staining with CD3 and TdT but negative immunostaining for CD4, CD8, CD19, and CD2. There are scattered clusters of dendritic cells showing positive staining for CD1a and S100 but negative for Langerin. The residual follicles stain partially for pax-5 and CD79a. The overall morphological and immunophenotypic features are of a primitive haematopoietic neoplasm with multilineage differentiation with predominant picture of AML.

### 5.1. Cytogenetic Analysis

We performed interphase cytogenetics fluorescence in situ hybridization (FISH) to ascertain the presence or absence of AML specific abnormalities. Cytogenetic/FISH studies were negative for AML panel specific abnormalities, and in our institute we perform the following probes panel: AML-ETO (DC,DF)/t(8;21)(q22;q22), PML-RARA (DC,DF)/t(15;17)(q22;q21), CBFB (DC, BAR)/inv (16)(p13;q22), CEP8/D8Z2, and MLL (DC,BAR)/11q23.

## 6. Discussion

 GS is a rare tumor formed by primitive myeloid cells at an extramedullary site. GS was first described by Burns in 1811 and named chloroma in 1853 [9, 10]. It is more of a localized tumor than a systemic disease. Although it is well recognized that granulocytic sarcoma can cause localized lymphadenopathy, widespread nodal involvement by AML, clinically mimicking non-Hodgkin's lymphoma, has been previously described in few reports in the English medical literature [2, 3, 6, 8]. Patients with granulocytic sarcomas are frequently asymptomatic; 50% of cases are diagnosed only at autopsy [11]. These tumors can involve any part of the body, either concurrently or sequentially. The most common setting for granulocytic sarcoma is disease progression in acute myeloid leukemia (73% of the patients) [6]. 

The present case report is in accordance with the previously reported similar studies [2, 3, 6, 8], in which GS presented with generalized lymphadenopathy. However in other studies [12] the central nervous system, subcutaneous tissues, and genitourinary system were the most common sites of disease, accounting for nearly 52% of all lesions, and in a study by Pui et al. [13], the skin and orbit were the most common sites. Even though it is known that granulocytic sarcomas can present with lymphadenopathy, it is not common. Thus, usually the diagnosis of GS is not entertained as a differential diagnosis for generalized lymphadenopathy, even in patients presenting with clinical picture suggestive of acute leukemia. 

The correct diagnosis is sometimes challenging due to variable morphology of GS and histological and radiological similarities to malignant lymphoma [14]. In our case the clinical presentation and radiological and FNA findings were suggestive of lymphoma which further highlights the importance of doing excision lymph node biopsy to reach a correct diagnosis. Moreover, the initial impression of this case was labeled as large cell lymphoma on immunohistochemistry. In a study done by Meis et al., 16 patients were diagnosed with granulocytic sarcoma without evidence of acute leukemia; twelve of these cases (75%) were initially labeled as large cell lymphoma [15]; there is a similar scenario in our case as the initial diagnosis was lymphoma. GS may precede the AML by months or years and can therefore be difficult to differentiate from lymphoma by clinical, radiologic, and even histopathological methods [11]. In such cases proper choice of an immunohistochemical panel including at least anti-CD43 or antilysozyme and special staining are necessary to reach accurate diagnosis. Use of more specific markers of myeloid disease, such as CD33, myeloperoxidase (MPO), CD34, and CD117, is necessary to establish the diagnosis. Other antibodies may be added depending on the differential diagnosis [16, 17].

In our case study, the initial impression was large cell lymphoma. But examination of peripheral blood, bone marrow and flow cytometry analysis were reported as AML with monocytic differentiations (FAB: M5) raising the suspicious of GS rather than Lymphoma. This issue highlights the importance of BM examination for the diagnosis of GS to assess the presence or absence of AML [18]. 

In brief, although rare, granulocytic sarcoma should be included in the differential diagnosis of generalized lymphadenopathy. The accurate diagnosis of this tumor needs to be aware of this disease; cooperation between clinician and pathologist and the application of proper immunohistochemical panel and special stains to detect the myeloid origin are important.

## 7. Conclusion

In summary, although it is well recognized that granulocytic sarcoma can cause localized lymphadenopathy, GS manifesting as multiple lymphadenopathy is a rare entity and bilateralism is very rare; it can mimic lymphoma cytologically and histologically. 

This tumor is often misdiagnosed as malignant lymphoma because of cytomorphologic and histologic similarities of the blasts to large cell lymphoma. A careful search for immature myeloid is a useful clue to the diagnosis accompanied with appropriate immunophenotyping.

## Abbreviations

GS: Granulocytic sarcoma
AML: Acute myeloid leukemia
BM: Bone marrow
FNA: Fine needle aspiration
FAB: French-American-British
MPO: Myeloperoxidase
TdT: Terminal deoxynucleotidyl transferase.
